# Comparative analysis of centrally mediated and inflammatory pain experiences amongst patients diagnosed with rheumatoid arthritis: A multimethods study

**DOI:** 10.1111/hex.14090

**Published:** 2024-06-05

**Authors:** Zoe Rutter‐Locher, Tom Esterine, Ruth Williams, Leonie S. Taams, Kirsty Bannister, Bruce W. Kirkham, Heidi Lempp

**Affiliations:** ^1^ Rheumatology Department Guy's and St Thomas' NHS Trust London UK; ^2^ Department of Inflammation Biology, Centre for Inflammation Biology and Cancer Immunology, Faculty of Life Sciences and Medicine, School of Immunology and Microbial Sciences King's College London London UK; ^3^ Department of Inflammation Biology, Centre for Rheumatic Diseases, Faculty of Life Sciences and Medicine, School of Immunology and Microbial Sciences King's College London London UK; ^4^ Central modulation of pain group, Wolfson Centre for Age‐Related Diseases, Guy's Campus King's College London London UK

**Keywords:** chronic pain, multiresearch methods patient involvement, rheumatology

## Abstract

**Background:**

The identification of pain originating from distinct biological processes may lead to individualised pain treatment. In this study, we aimed to explore the pain experiences of patients with rheumatoid arthritis (RA), differentiating between those predominantly exhibiting features of peripheral inflammatory versus centrally mediated pain.

**Methods:**

Through a multimethods approach we (i) quantitatively analysed the differences in pain descriptors between patients diagnosed with RA experiencing peripheral inflammatory and centrally mediated pain, utilising the Short Form‐McGill Pain Questionnaire which includes the pain visual analogue scale (VAS) and (ii) qualitatively explored their subjective pain experiences grounded in the biopsychosocial model, commonly applied in chronic pain.

**Results:**

Participants with centrally mediated pain reported higher pain scores on the VAS, used a wider range of pain descriptors, and a higher proportion selected each descriptor compared to those with inflammatory pain (*p* < .001). The qualitative analysis revealed the centrally mediated pain group's experiences were overwhelming and relentless, struggling to precisely articulate the nature of their pain. In contrast, individuals with inflammatory pain expressed their pain in more tangible terms and shared their adaptive and coping strategies. Importantly, both groups revealed the substantial psychological, functional and social impacts of their pain, highlighting the often ‘invisible’ and misunderstood nature of their symptoms.

**Conclusion:**

This study has gained a deeper insight into the pain experiences of patients living with RA, particularly in differentiating between centrally mediated and inflammatory types of pain, potentially facilitating a more individualised approach to pain treatment.

**Patient Contribution:**

Patients actively participated in the study conception and design. This engagement includes collaboration with key stakeholders, such as members of the National Rheumatoid Arthritis Society and Patient Research Partners (PRPs), who provided continuous feedback and guidance throughout the research process. Specifically, the qualitative element was coproduced with two PRPs, who were involved in co‐leading the focus groups and data analysis.

## 1 INTRODUCTION

Pain is the primary reason patients with inflammatory arthritis seek medical care.^1^ Recognised as a key symptom by patients,^2^ pain is integral to disease activity assessments and is highlighted as the foremost patient‐reported outcome by the American College of Rheumatology's Pain Management Task Force.^3^ The impact of pain extends beyond physical discomfort, influencing disease management, quality of life and social interactions,^4^, ^5^ and affects emotional wellbeing, with clear links to fatigue, depression and anxiety.^6^, ^7^, ^8^

Pain in rheumatoid arthritis (RA), arising from joint inflammation, has been characterised as a diffuse, burning ache that can range from moderate to severe intensity, typically fluctuating between flare‐ups and periods of remission.^9^ There is a growing acknowledgement that a significant proportion—up to 40%—of chronic pain in RA stems not from ongoing inflammation but from a dysregulated nervous system.^10^, ^11^ This dysregulation can be due to peripheral sensitisation or abnormalities in central processing within the spinal cord or brain, leading to increased pain sensitivity (hyperalgesia) and pain triggered by external stimuli that do not usually provoke pain (allodynia).^11^, ^12^ The degree to which pain is caused by dysregulation of the nervous system will vary between patients, with some experiencing predominantly inflammatory pain, some more centralised, but also many that have varying degrees of both. Identification of the primary pain driver in an individual is crucial, as each may require a different therapeutic approach.

The painDETECT questionnaire identifies neuropathic pain components such as ‘burning’, ‘electric shocks’ and ‘tingling or prickling’, alongside the pain course pattern and radiation of pain.^13^ Due to the similar symptom profile of neuropathic pain and centrally mediated pain, the painDETECT questionnaire was used to identify centrally mediated pain in RA research.^14^, ^15^, ^16^ Fibromyalgia, the prototypical central sensitivity syndrome, is thought to involve similar central mechanisms.^17^ There is growing interest in the role of quantitative sensory testing to identify abnormalities in specific pain processing pathways.^12^ However, the application of these questionnaires and tests in everyday clinical practice remains limited. The individual and multidimensional nature of the pain experience means that personalised pain descriptors may communicate pain more accurately than these questionnaire or experimental measures.^18^, ^19^ Further understanding of the pain experience in patients with peripheral inflammatory versus centrally mediated pain may help clinicians provide an individualised approach to pain treatment.

Various disease models have been applied to chronic pain,^20^, ^21^ and the biopsychosocial model stands out as a relevant and comprehensive approach to understanding pain in RA.^22^ First conceptualised by Engel in 1977,^22^ this model transcends the traditional view that pain is solely a biological phenomenon driven by physiological pathology but that psychological and social factors are also integral in shaping an individual's experience of pain. Psychological factors may include emotional distress, fear and coping strategies while social factors may include cultural aspects, social isolation and socioeconomic features. This holistic perspective emphasises that chronic pain is a multifaceted symptom, influenced by a complex interplay of biological, psychological and social dimensions.^21^

In this study, we aimed to explore the pain experience of patients with RA, distinguishing between those predominantly exhibiting features of peripheral inflammatory versus centrally mediated pain. Our methodology was twofold, employing a multimethods approach. First, we quantitatively analysed the differences in pain descriptors provided by patients with RA with peripheral inflammatory and centrally mediated pain, using the Short Form (SF)‐McGill Pain Questionnaire and VAS.^23^ Second, we applied a qualitative approach, aiming to understand the subjective pain experiences of patients with RA and comparing the accounts of both groups within the context of the biopsychosocial model.

## 2 METHODS

The concept of this study occurred as a result of discussions held at a workshop attended by academics, clinicians and patient research partners (PRPs) in 2018. Members of the National Rheumatoid Arthritis Society have been actively involved in the study design, including questionnaire choice. PRPs have provided continuous feedback regarding study design for both the quantitative and qualitative elements of the study, such as tolerability of elements of the study visit and feedback on the focus group topic guide.

### 2.1 Patient recruitment

A purposive sample^24^, ^25^ of patients were invited from a larger body of participants from the study ‘Pain phenotypes and their underlying mechanisms in Inflammatory Arthritis (PUMIA)’.^26^ Inclusion criteria were adults with a diagnosis of RA, reporting current numerical rating scale pain ≥3 on a 0–10 scale, able to communicate effectively in English and fulfil criteria for predominantly peripheral inflammatory or centrally mediated pain as below. The participants had previously completed extensive sensory assessments including the painDETECT questionnaire^13^ and evaluations based on the revised 2016 criteria for fibromyalgia^27^ for centrally mediated pain, and targeted musculoskeletal ultrasound (MSK US) of 14 joints,^28^ as a measure of peripheral inflammation.

The individuals were stratified into two distinct groups based on their type of pain: (i) predominantly peripheral inflammatory pain and (ii) predominantly centrally mediated pain. The identification for the peripheral inflammatory pain group required active synovitis evidenced on MSK US (with a minimum power Doppler score of 1 in any of the 14 joints examined), a painDETECT score of 18 or below and did not fulfil fibromyalgia criteria. The centrally mediated pain group included individuals with a high painDETECT score exceeding 18, fulfilment of the fibromyalgia criteria and no active synovitis on MSK US (no power doppler signal in any of the 14 joints examined). The selection process for the qualitative part also included a balance of age, gender and ethnicity to reflect the diverse experiences of pain across these demographic characteristics. All provided written informed consent and the study was conducted with the approval of the Bromley Research Ethics Committee and the Health Research Authority (REC 21/LO/0712, 8/12/2021).

### 2.2 Quantitative data collection

All participants completed the SF McGill Pain Questionnaire.^23^ This questionnaire asks participants to rate (as none = 0, mild = 1, moderate = 2, severe = 3) 11 sensory descriptors and four affective descriptors of their pain, followed by a visual analogue scale (VAS) for present pain intensity.^23^

### 2.3 Quantitative data analysis

Mean and standard deviation (SD) of the sensory/affective/VAS was compared in the peripheral versus central group using unpaired *t*‐test. Frequency of descriptor chosen (using a cut‐off value of at least 2, moderate) was then compared in the peripheral versus central group of participants.

### 2.4 Qualitative data collection

A draft focus group schedule was developed following a review of the literature,^9^, ^18^, ^29^ the experiential knowledge of the research team and suggestions from the PRPs. To ensure relevance, comprehension and clarity of the questions, including timing and research burden, the focus group schedule was piloted with one patient by Z. R. L. The then refined focus group guide consisted of three key questions: description of their pain, impact on their daily life and what message they have to others who do not experience long‐term pain (see Supporting Information Material).

Two focus groups were held, one for each pain group, remotely via Microsoft TEAMS ensuring accessibility and convenience for all participants. The audio‐recorded focus groups were co‐led by H. L. and one male PRP (T. E.) and lasted approximately 1 h. Field notes were made during each focus group. Researcher H. L. is a female medical sociologist with extensive experience in leading focus groups and before the focus groups had provided training to the PRP. Neither the researcher nor the PRP had any prior contact with the participants or were involved in their regular care. This ensured a neutral starting point without any preconceptions or biases from either side. Four of the invited participants from the centrally mediated pain group were unable to ultimately attend the focus group due to health reasons (*n* = 2) and other personal commitments (*n* = 2). One member of the peripheral inflammatory pain focus group could not attend on the designated date and was subsequently interviewed separately. His information was then included into the relevant subgroup for data analysis.

### 2.5 Data analysis

Each focus group/interview was transcribed verbatim by an external professional transcribing agency, and the content double checked for accuracy by Z. R. L. Content data analysis^30^, ^31^ was conducted assisted by NIVIVO 14 (qualitative computer software programme^32^). Initial coding was conducted independently by researcher Z. R. L. and PRP, T. E., by repeatedly reading the interview text. These preliminary codes were then cross‐checked and discussed with co‐authors H. L. and PRP, R. W. to ensure relevance and validity. Subsequently, Z. R. L. identified themes through clustering of related codes. This collaborative approach resulted in a consensus to delineate eight subthemes that aligned with the three dimensions of the biopsychosocial model, which we used as a framework (see Section 3). Data saturation was reached following two focus groups.

### 2.6 Data validation

The data analysis was subject to a range of validity checks: (1) a draft focus group topic guide was piloted with one patient to ensure the questions were relevant and comprehensive (2) initial codes were identified independently by two authors (Z. R. L., PRP T. E.) and then cross‐checked by two further authors (H. L., PRP R. W.) (3) the accounts in Section 3 from all the participants are included (4) ‘Deviant’ accounts^33^ are incorporated into the results section to achieve a balanced reporting. Member checking^34^ was not employed to avoid overburdening participants given their level of pain.

## 3 RESULTS

### 3.1 Quantitative results

From a total of 113 patients, 29 participants fulfilled criteria for the inflammatory group and 19 participants fulfilled criteria for the central group, totalling 48 participants. The mean age was 54 (SD = 15), 38 (79%) were female and the mean disease duration was 6.1 years (SD = 9.8).

Individuals with centrally mediated pain reported significantly higher mean scores for pain VAS and for each class of descriptor (sensory, affective, total) compared to those with inflammatory pain (Table 1). According to the Melzack approach,^23^ for a descriptor to be considered specific for a group of patients, the descriptor must be chosen by at least a third of the subjects. 14/15 descriptors fulfilled that criterion in the central group and 5/15 in the peripheral inflammatory one. In addition, a greater proportion of participants in the central group were reporting each pain descriptor than in the peripheral group (Table 2). In both groups ‘aching’ was a key descriptor, in the top three rated. However, in the central group ‘tiring‐exhausting’ predominated with ‘shooting’, ‘cramping’ and ‘heavy’ other key descriptors not identified in the inflammatory one (Table 2).

**Table 1:** Results of the SF‐McGill Pain Questionnaire.

	Central	Inflammatory	*p* Value
Sensory	21.3 (5.3)	11.7 (6.7)	<.001
Affective	7.6 (3.1)	2.7 (2.6)	<.001
Total	28.9 (7.9)	14.4 (8.5)	<.001
VAS	6.8 (2.0)	4.1 (1.8)	<.001

*Note*: Eleven sensory descriptors and four affective descriptors were assigned a score of 0 (none) to 3 (severe) by each participant, followed by a VAS for present pain intensity. Mean (SD) shown scores compared between those with central and inflammatory pain, using *t*‐test.

Abbreviations: SF, Short Form; VAS, visual analogue scale.

**Table 2:** Key descriptors identified by participants with central versus inflammatory pain.

Central (*n* = 19)	Inflammatory (*n* = 29)
Tiring‐exhausting (95%)	Aching (62%)
Aching (89%)	Tender (55%)
Shooting (84%)	Sharp (48%)
Cramping (84%)	Tiring‐exhausting (41%)
Heavy (84%)	Hot‐burning (34%)
Tender (84%)	
Sharp (79%)	
Throbbing (74%)	
Stabbing (74%)	
Hot‐burning (58%)	
Sickening (58%)	
Cruel‐punishing (58%)	
Fearful (53%)	
Gnawing (47%)	

*Note*: 14/15 descriptors were used by at least 1/3 participants in the central group and 5/15 in the peripheral inflammatory group. Frequency that the descriptor was used by participants in each group is shown in parenthesis ‘()’.

### 3.2 Qualitative results

#### 3.2.1 Focus group sociodemographic information

Seven participants agreed to take part. Both focus groups included participants from diverse ethnic heritage and mainly females with high pain levels (Table 3).

**Table 3:** Sociodemographic information of focus group participants.

	Central pain group	Inflammatory pain group
Age	54 (4.6)	51 (11.5)
Gender	Three female	Three female and one male
Ethnicity	One White British, one White‐other white background, one mixed‐white and Black Caribbean	Two White British, one Black African, one Black Caribbean
Employment	One full‐time, two unemployed	Three full time, one unemployed
Pain score	7.7 (0.6)	6 (3.2)
Disease duration	16.7 (9.5)	9.5 (10.7)

#### 3.2.2 Key themes and subthemes

**Figure 1:**
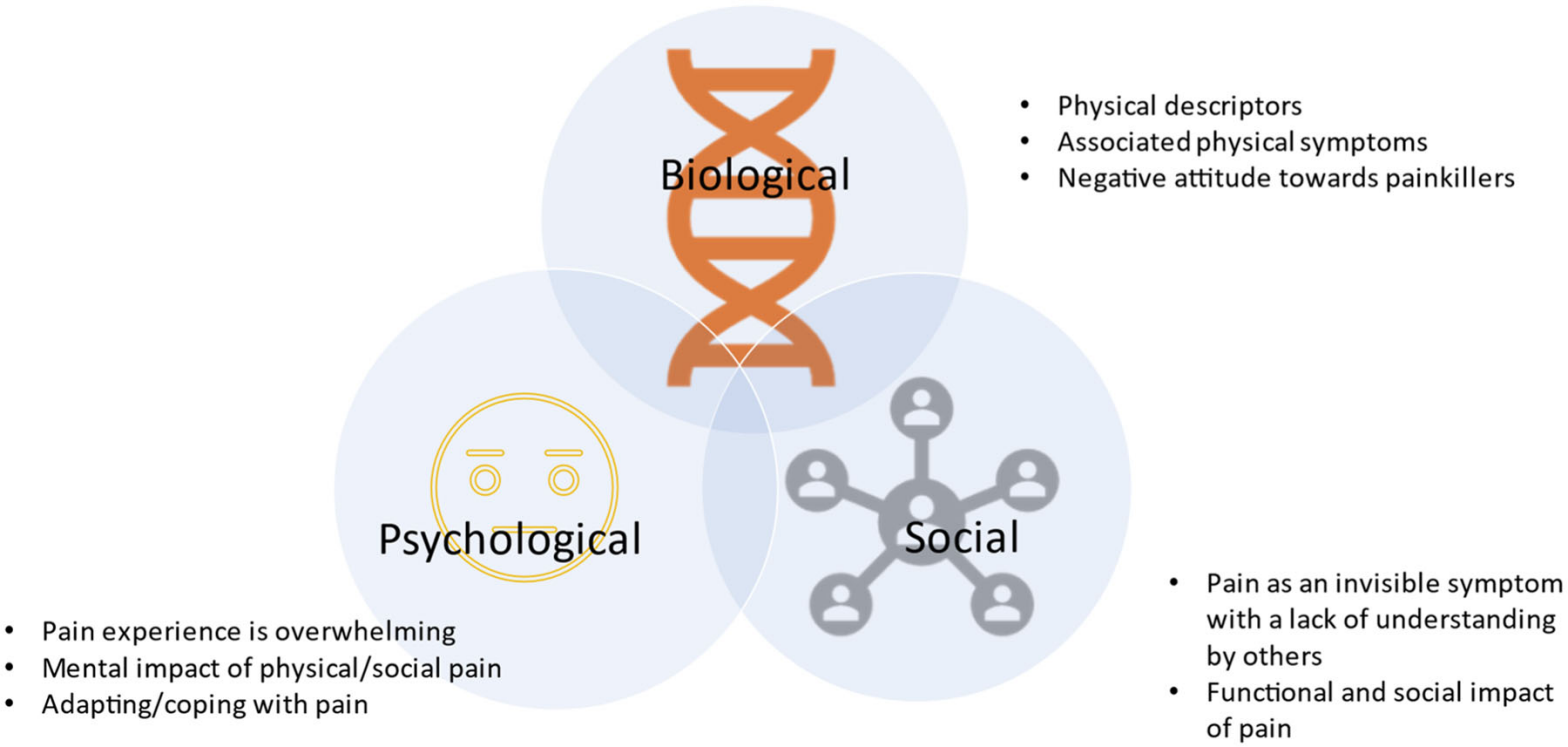
Biopsychosocial model with eight identified subthemes.

We present the key themes based on the biopsychosocial model,^22^ and each includes 2–3 subthemes as illustrated in Table 4 and Figure 1.

**Table 4:** Key themes and subthemes based on the biopsychosocial model.

Key theme	Subtheme
Biological dimension	Physical descriptors
	Associated physical symptoms
	Negative attitude towards painkillers
Psychological dimension	Mental impact of physical/social pain
	Pain experience is overwhelming
	Adapting/coping with pain
Social dimension	Pain as an invisible symptom with a lack of understanding by others
	Functional and social impact of pain

Below, we describe the themes and subthemes, highlighting the similarities and differences between accounts identified in the ‘peripheral inflammatory’ versus ‘centrally mediated’ pain groups. We have included illustrative accounts in italic within the paragraph and identify participants by focus group (I = inflammatory group, C = centrally mediated group) followed by number, for example, I1.

### 3.3 Key theme: Biological dimension

#### 3.3.1 Physical descriptors of pain

Participants from both focus groups emphasised the intensity of pain associated with their condition, although using distinct descriptors to articulate their experiences, sharing phrases such as ‘My pain is unbearable’ (C1), and ‘the pain sometimes is just so intense, that you just want to give up for the day’ (I1).

People with centrally mediated pain described their discomfort as severe and relentless, often leading to profound exhaustion post‐physical activity, ‘And so yes … there is something every day, some kind of pain every day’ (C2). They reported having a high pain threshold, ‘so I might describe my pain as like a 6 or a 7 [on the VAS] but to somebody else …. having the pain that I was having …they would say it was a 10’ (C2).

Conversely, individuals with inflammatory pain described their pain in more tangible terms, characterised, for example, as aching, simmering or a hot, burning sensation. They detailed the pain as a feeling of tenderness and stiffness with phrases, such as ‘the pain I experience is stiffness in my fingers’ (I2) as well as the sensation of swelling in the joints. This group differentiated between a constant, widespread low‐level baseline pain ‘very low level pain, everywhere, like every joint hurt, but not that badly’ (I4) and the acute discomfort stemming from inflammatory flare‐ups ‘nine times out of ten when I'm actually having a really nasty flare, I'm fatigued, I'm feeling fluey, I feel tearful, I feel a bit depressed, you know, I'm struggling to do the most simplest of tasks’ (I2).

Individuals in the inflammatory group also noted the systemic nature of their pain, similar to flu‐like symptoms, muscle aches, a sense of inflammation throughout the body and pain that migrated between joints.

##### Timing of pain

Individuals experiencing central pain reported heightened discomfort during the night, particularly when they were relaxed, and consistent pain throughout the day, ‘the pain varies but during the night as soon as I relax the pains come in in the back’ (C3). In contrast, those with inflammatory pain noted a stark increase in their pain levels in the morning, ‘I'd say from morning till night, you go through different experiences, you might be bad in the morning, come the afternoon you're walking around and you're fine’ (I1).

##### Triggers of pain

In both focus groups, participants identified emotional stress and poor sleep resulting in physical fatigue as a major trigger for their pain. One participant with central pain shared ‘Yeah. When I'm really tired … that really tends to make me feel much worse …’ (C2) and another with inflammatory pain said, ‘triggers are a bad night's sleep …. and also a real big trigger is particularly bad stress’ (I4).

In addition, those with central pain described how both physical exercise and staying stationary activates their pain with one participant explaining ‘if I travel in the car for the long journey, when I arrive [to the] destination I'm paralysed’ (C3). Weather changes such as low pressure and rain were also mentioned as pain prompts one participant reporting ‘Even in the summer if there is a change of weather, that there's a thunderstorm, I know long before the weathermen knows that it's gonna happen’ (C2).

##### Alleviators of pain

Many shared that varying temperatures, such as cold or heat, played a role in alleviating their pain. In contrast to one another, while remaining stationary tended to ease the discomfort of those with central pain (Interviewer: ‘Can you tell me what relieves the pain? Is there anything?’, Respondent: ‘I don't move’ [C1]). Individuals with inflammatory pain found relief in movement and using their joints; ‘exercise makes [the pain] better’ (I3).

#### 3.3.2 Associated physical symptoms

Participants from both focus groups emphasised the significant impact of pain in disrupting their sleep. One individual stated, ‘I'm not getting full sleep, every hour I'm getting up, every hour I'm twisting turning, and if I have 2 hours sleep, I say it's a blessing’ (C1). Another echoed this sentiment with a longing for relief: ‘Oh, what I'd give for a good night's sleep [laughs], just one good night's sleep. You can't lay on your side because of the pain, it's just so painful, and then you turn onto the other side and it affects the other joints, because you're putting your weight on the side’ (I1). Furthermore, they shared their debilitating physical fatigue and stiffness. Two participants described ‘the fatigue feels like your whole body, your brain, everything is, has just been taken over by this feeling of being generally, just exhausted’ (I2), ‘It's hard to move. I'm like a tin man, a tin woman’ (C1). Specifically, those with central pain described a persistent sense of exhaustion even after sleep and waking unrefreshed. One described this experience saying ‘I say it's like running on an empty battery all day, all time …. you don't charge up …. you don't go to bed and feel refreshed in the morning you get up and you're just as exhausted as you went to bed’ (C2).

#### 3.3.3 Negative attitude towards painkillers

Individuals experiencing central pain mentioned that painkillers offered minimal relief for their symptoms. Additionally, they highlighted the adverse side effects of these medications, such as nausea and drowsiness, compounded their discomfort; ‘I'm floating on the ceiling but not doing the job it's meant for which is to take [away] the pain’ (C2). Those with inflammatory pain did not discuss attitudes towards painkillers.

### 3.4 Key theme psychological dimension

#### 3.4.1 Mental impact of physical/social pain

Both group members described the psychological impact of their pain, but with slightly different emphasis. Those with central pain expressed worries about their functional disability and the looming expectation of recurring pain sensations. One individual captured this anxiety, saying, ‘even now I was worried about doing this because of the way my hands are (C2).’ In contrast, participants with inflammatory pain did not share this particular psychological impact.

Frustration was a common feeling among both groups, but its sources varied. Those with central pain felt frustrated due to their reliance on assistance from family and friends. Meanwhile, those with inflammatory pain expressed annoyance related to their physical constraints and dependency on healthcare services, including regular hospital visits and medication routines. One person from this group conveyed their exasperation: ‘I feel very, very fed up by the whole process. Having to get myself up to hospital to sit and talk to doctors about my condition, to repeat the same story over and over again’ (I2). Those with inflammatory pain described embarrassment in front of their friends and family about their pain and reduced physical function coupled with a loss of self‐confidence, ‘people [were] kicking off, fighting on the top deck of the bus, and I thought, oh no, so I went down the stairs, then I didn't feel particularly, you know, physically robust … and the bus jolted and it just pulled my arm and I thought what am I just doing this for, I should just be in bed. But then you hate that about yourself, kind of thing’ (I4).

Low mood as a result of persistent pain was a significant psychological impact identified by both groups. One individual described how external stressors easily compounded their mental health challenges: ‘Because with everything else going on with the pain, everything, with the tiredness, every little thing from the outside it affects my mental health, so [I am] a bit stressed and depressed quite easily because of everything already going on’ (C3). Additionally, those with central pain noted that low mood was a comorbidity even before their diagnosis of RA with one sharing, ‘I had depression and everything beforehand so this on top. It's just been one thing after another with health’ (C2).

One person with inflammatory pain highlighted how chronic pain amplified the distress caused by everyday adversities: ‘My life is very pleasant overall, like I'm under no illusions, but obviously there are things that are miserable and depressing anyway, and then they weigh on you more heavily …you sort of think … come on mate, I've already got enough going on, don't add to it’ (I4).

Lastly, participants with inflammatory pain expressed grief over the loss of their previous active lifestyles. They shared feelings of being unjustly deprived, as one mentioned: ‘But, you know, it's not fair, I feel like I've been robbed of something, I've been robbed of the opportunities that I used to have, I grieve the old [name] that could do the things that she used to be able to do’ (I2) and another commented, ‘I [used to] have [a] very dynamic life, and now [that has] stopped, completely stopped … it's very depressing for me’ (I3).

#### 3.4.2 Pain experience is overwhelming

All of those experiencing central pain emphasised the all‐consuming aspect of their condition, a sentiment not echoed by those with inflammatory pain. One described how the pain had taken over her life (C1) and the intense additional mental exhaustion, stating, ‘It's mentally exhausting as well because I have to prepare and I have to make sure I'm only [going] out for like [a] couple of hours’ (C1).

#### 3.4.3 Adapting/coping with pain

Participants with inflammatory pain shared various adaptive strategies and coping mechanisms they use to manage their pain, in contrast to those with central pain. They spoke about acceptance and maintaining a positive mindset despite their challenges. One person reflected this attitude: ‘I know I have to live with it, and I've got no choice, and each day I do my best to try and be positive’ (I2). Another shared their path to acceptance: ‘And there is a point where you can just sort of go, like take a deep breath and go, right …. oh get over it kind of, you know …. then eventually you think oh I'm just going to get on with it now and you just do and it's not actually that bad’ (I4).

Some individuals also discussed the necessity of ‘pushing through’ pain to maintain normal activities and engage with clinical services for improvement. The fluctuating severity of their pain, with some days feeling better than others, also brought them hope, as one expressed: ‘Sometimes you wake up and you're like, oh good, this is a good day, today is a good day’ (I1).

In terms of practical adaptations, one participant talked about using pillows for comfort in bed, along with massage and exercise (I2). Another mentioned utilising specific tools to reduce the strain on their joints, like a special spanner for opening bottles and choosing straight glasses over heavier beer mugs (I4).

Adjustments were applied to their lifestyles, with modifications in dressing due to comfort needs (‘I don't wear fashion shoes people, I'd love to, but I don't (I2)’) and alterations in work patterns to accommodate joint discomfort. Additionally, participants stressed the importance of recognising their limits, as one said, ‘I still do go out, not as often, and I have to admit that I'm learning to tell myself if you don't feel well don't push yourself’ (I2).

### 3.5 Key theme social dimension

#### 3.5.1 Pain as an invisible symptom with a lack of understanding by others

Members from both focus groups highlighted that pain is an invisible symptom and emphasised the challenge of explaining their discomfort to others. They mentioned their experiences of a hidden symptom that is hard to quantify and often misunderstood by others, for example, family, friends and work colleagues. One illustrated this by saying, ‘in my case it's very hidden, and I don't walk with an aid, once I've got going, once I'm out of the house and got moving I walk quite normally’ (I4). Another alluded to the discrepancy between their outward appearance and what they truly felt: ‘I might look okay and you take for granted that I'm doing okay today but you don't understand that sometimes even if I'm not saying I'm in pain I'm in agony’ (C2).

The difficulty of describing their pain to those who have never experienced such intense discomfort was a common theme. One individual highlighted: ‘I wouldn't know how to express to people who have no idea about it, hit yourself with a hammer on your hand, there you go, try that one’ (I1).

They acknowledged the inherent complexity in understanding another person's pain sensation, as expressed in statements like ‘I can't ever understand another person's pain, I may be able to have an insight somehow, but I can never’ (I2) and ‘it's hard for people to imagine how you are feeling if they haven't got this amount of pain. It's incredibly hard for somebody to understand’ (C2).

In light of these challenges, participants with central pain specifically spoke about the need for patience and understanding from others. As one requested, ‘please be patient and please try to understand how I'm feeling’ (C1).

#### 3.5.2 Functional and social impact

Participants from both groups highlighted the functional and social impact of pain on their lives. They described the exhausting and painful nature of routine activities, such as showering, with one person saying ‘to have a shower is exhausting, it's painful, so I have to have a stool’ (C1). They also described their inability to partake in their hobbies, especially those involving their hands such as knitting or crafts. Of particular pertinence were descriptions of their inability to engage with their children or grandchildren, stating ‘I could not lift her up to put her on the swings, I could barely push her …. I sit on the bench and watch. You want to get up and be with them …. it's so heartbreaking … it just is awful’ (I1). Furthermore, those with inflammatory pain discussed the need to adjust or even abandon their work due to joint discomfort, as one shared, ‘I've had to actually adjust my working patterns because of the joint discomfort’ (I2), while another had to give up work entirely (I3).

Participants highlighted the significant impact on their ability to socialise with their friends. For some this was because they were too tired to socialise, ‘not in the right mental state’ (C3) or ‘not feeling up to it’ (I4). Another expressed the constant presence of pain during social interactions: ‘I can't get up and dance, I can't interact with people, I can't, because … the pain is always at the forefront of your brain, so you're just like, when can I leave, so I don't, I've literally given up on my social life’ (I2). The need for advance planning for social events was also a constraint, as highlighted by one participant: ‘if you phone me now and say ‘oh [name] now let's go out’, I will say ‘I'm sorry, I can't’ (C1). This limited ability to socialise led to fears of social isolation, with one participant voicing a concern that friends might eventually stop inviting them out, saying, ‘one day people will stop asking, because oh she never goes out anyway, so they'll stop asking’ (I2).

As discussed, the summary of our key findings are represented within the biopsychosocial model, resulting in eight subthemes (Figure 1). Table 5 presents a summary of key similarities and differences in themes/codes identified between those with predominantly inflammatory versus centrally mediated pain.

**Table 5:** Similarities and differences in subthemes/codes in those with predominantly peripheral inflammatory versus predominantly centrally mediated pain.

	Predominantly peripheral inflammatory pain	Predominantly centrally mediated pain
Biological	Severe intensity of pain	
	Emotional stress and poor sleep triggers for pain	
	Associated stiffness	
	Associated fatigue	
	Pain leading to sleep disruption	
	Tangible pain descriptors (e.g., aching, hot, simmering, tenderness)	Relentless pain
	Differentiation between constant widespread low‐level discomfort and acute inflammatory flare pain	Pain leads to profound exhaustion
	Systemic nature of their pain likening it to flu‐like symptoms/muscle aches	Mental exhaustion and waking unrefreshed
	Pain worse in the morning	Pain worse at night and constant throughout day
		Limited relief from painkillers with significant adverse effects
Psychological	Low mood because of pain	
	Frustration at dependency on hospital services	Worry about functional disability
	Embarrassment in front of family and friends	Worry about expectation of recurring pain sensation
	Grief over loss of previous active life	Frustration at reliance on family and friends
	Use of adaptive strategies and coping mechanisms to manage pain	Low mood as pre‐existing comorbidity
		Pain experience is overwhelming
Social	Pain as an invisible symptom with a lack of understanding by others	
	Difficulty in describing pain to others	
	Inability to partake in activities of daily living, hobbies and family events	
	Significant impact on ability to socialise with friends	

## 4 DISCUSSION

The multimethodological approach of the study has provided detailed insight into the extensive impact of pain in patients diagnosed with RA, augmented by the distinction between centrally mediated and inflammatory pain. The quantitative data revealed through the SF‐McGill questionnaire how the pain experience is perhaps more diverse and intense in those with centrally mediated pain than those with inflammatory pain. The multidimensional experience of pain seems to encompass physiological, sensory, affective and cognitive components.^35^ While tools such as the VAS primarily measure self‐reported pain intensity, the Short‐Form McGill Pain Questionnaire offers a more comprehensive evaluation, capturing both sensory and affective dimensions of pain.^23^, ^36^ In this study, the average scores for both sensory and affective pain descriptors in the inflammatory group were similar to those observed in previous studies of patients with RA.^37^, ^38^ However, interestingly, the higher average scores and the pain descriptors in the central group of this study reflected findings typically associated with fibromyalgia.^37^

In qualitative accounts, centrally mediated pain was described as more generalised, unrelenting pain with exhaustion being a particularly dominant symptom. Those with centrally mediated pain frequently struggled to articulate their pain precisely, resorting to metaphors, a phenomenon well‐documented in pain literature.^37^, ^38^ In contrast, individuals with inflammatory pain tended to express more tangible descriptors for their pain, in keeping with the underlying pathophysiology of joint and systemic inflammation. These clinically relevant descriptors can offer promising additional insights for assisting clinicians to distinguish between centrally mediated and inflammatory pain in clinical practice, thereby facilitating targeted pain treatment. Their validity as a definitive classification tool remains to be established, but in the meantime, these descriptors serve as potential candidates that can assist clinicians in acknowledging and recognising the diverse pain types associated with RA.

Historically, pain was understood to correlate directly with the underlying biological pathology, and psychological factors were considered only in the absence of identifiable physical causes.^39^ However, substantial evidence has now significantly broadened this perspective, highlighting the equal importance of biological, psychological and social determinants in understanding pain.^21^, ^40^, ^41^ This holistic view is reflected in the biopsychosocial model of chronic pain.^22^ As well as the identification of specific pain descriptors, recognising the need to assess the interplay between biological, psychological and social factors in each individual's experience of pain has important implications for stratified pain treatment, setting the path for more effective pain management.

Psychosocial factors significantly influence long‐term pain outcomes and treatment responses.^42^ The bidirectional relationship between persistent pain and low mood is well‐documented, evident in longitudinal studies of musculoskeletal and other chronic pain conditions^43^, ^44^ and indeed, both individuals with inflammatory and centrally mediated pain reported a substantial impact of pain on their mood. Furthermore, studies have shown that pre‐existing psychological conditions can predispose individuals to chronic pain.^45^, ^46^ For instance, a patient with central pain in this study cited a previous diagnosis of depression, raising the possibility that such psychological distress might have predisposed her to the development of chronic pain in the absence of inflammation. Overcoming psychological distress early could then potentially play a role in aiming to prevent the development of chronic pain^47^ and emphasises the need for early screening of mental health comorbidity.

As low mood has been associated with heightened pain, the use of coping strategies by patients has been linked to both lower pain levels and decreased sensitivity to experimental stimuli.^48^, ^49^, ^50^, ^51^ Furthermore, studies investigating the use of interventions focusing on positive attitudes and behaviours, for example, personalised positive psychology exercises,^52^ suggest they may be beneficial in managing long‐term pain.^52^, ^53^ Individuals in the inflammatory group shared various adaptive strategies and coping mechanisms. These included problem‐focused strategies to mitigate the physical impact of pain, such as using pillows for comfort in bed, and emotion‐focused approaches to manage the emotional responses to pain, emphasising acceptance and maintaining a positive mindset.^51^

In contrast, those with centrally mediated pain described an overwhelming sense of pain, possibly indicating a tendency towards catastrophising. Pain catastrophising is a cognitive and emotional response characterised by an exaggerated perception of pain, persistent preoccupation with pain and a sense of helplessness in its management.^54^ This response is associated with increased pain intensity, greater disability, psychological distress and notably, it is a major risk factor for poor treatment outcomes.^44^, ^55^, ^56^ Additionally, significant anxiety, including the worry about impending pain, was a common theme among those with central pain. This is consistent with the framework underlying cognitive‐behavioural techniques used in chronic pain, that pain catastrophizing and fear of pain can lead to avoidance behaviours and consequent physical deconditioning.^57^

The presence of supportive social networks are associated with positive health outcomes, such as less perceived pain and improved physical and social functioning.^41^ For instance, presence of a supportive and understanding partner has been shown to not only result in less pain but also improved quality of life and reduced psychological distress.^58^, ^59^ A common key theme expressed by both focus groups was the challenge of pain being an ‘invisible’ symptom, often leading to misunderstandings by others. This phenomenon is well‐recognised and linked to increased pain severity, psychological distress and poorer social functioning.^60^, ^61^ Detrimental for patients is when facing ‘discounting’ or the denial and patronisation of their pain experience, which can result in undermining the legitimacy of their pain.^62^ A recent study highlighted that pain invalidation can affect both individuals with fibromyalgia and RA. Interestingly, patients reported more instances of their pain being discounted by family members and colleagues than by medical professionals.^60^

## 5 STRENGTHS AND LIMITATIONS OF THE STUDY

To our knowledge, this study is the first to qualitatively explore pain experience in carefully selected subjects that may experience distinct forms of pain. A strength of this study was the use of stringent criteria on validated questionnaires and MSK US to distinguish individuals with predominantly inflammatory pain from those with centrally mediated pain. The multimethod approach with key involvement of patients, from the initial conception and design phases through to data analysis, ensured that their perspectives were considered throughout the study. The qualitative arm of the study complemented the quantitative findings, namely providing very rich accounts that led to invaluable insight of patient's daily experiences of pain. Specifically, the incorporation of focus groups not only enabled the patient partner to co‐lead the interviews but also facilitated a collaborative atmosphere where participants could build on one another's responses, resulting in generation of ideas which may have not been identified in an individual interview. Although we acknowledge the modest sample size, a recent study by Malterud et al alluded to the concept of ‘information power’, whereby the more information the sample holds, the lower the number of participants is needed,^63^ highlighting rich data can be gained from modest samples.

The limitations related to the recruitment, namely, while we successfully included participants from diverse ethnic backgrounds, we recruited only one male participant for the focus groups. Although RA does have a strong female predominance, with women three times more likely than men to develop the condition,^64^ this gender imbalance is significant, as it is well established that pain experiences can differ between females and males.^65^ Women are more likely to experience persistent and severe pain and demonstrate greater sensitivity to experimental stimuli.^66^ These differences may be attributed to variations in spinal and supraspinal pain processing mechanisms, as well as psychosocial factors, such as societal norms influencing males to report higher pain tolerance.^67^ Finally, it is important to highlight that individuals categorised as having centrally mediated pain, on average, had a longer disease duration compared to those experiencing inflammatory pain. This raises a challenge in distinguishing whether the variation in pain experiences is solely attributed to the type of pain or if the duration of the disease also played a role.

## 6 CONCLUSION

This study has gained a deeper insight into the pain experience in patients with RA, particularly in differentiating between centrally mediated and inflammatory types of pain. The process of identifying key pain descriptors in these distinct pain groups, coupled with an in‐depth examination of the psychological and social impacts of these pain experiences has the potential to facilitate a more individualised approach to pain treatment in the future.

## AUTHOR CONTRIBUTIONS

**Z. Rutter‐Locher**: Conceptualisation; formal analysis; writing—original draft. **T. Esterine**: Methodology; formal analysis; writing—review and editing. **R. Williams**: Formal analysis; writing—review and editing. **L. S. Taams**: Supervision; writing—review and editing. **K. Bannister**: Supervision; writing—review and editing. **B. W. Kirkham**: Conceptualisation; supervision; writing—review and editing. **H. Lempp**: Conceptualisation; methodology; writing—review and editing; formal analysis.

## CONFLICT OF INTEREST STATEMENT

The authors declare no conflict of interest.

## ETHICS STATEMENT

The study was conducted with the approval of the Bromley Research Ethics Committee and the Health Research Authority (REC 21/LO/0712, 8/12/2021).

## Supporting information

Supporting information.

## Data Availability

The data that support the findings of this study are available from the corresponding author upon reasonable request.
