# Supplementary material for: Comparative analysis of centrally mediated and inflammatory pain experiences amongst patients diagnosed with rheumatoid arthritis: A multimethods study
Source: Health Expect. 2024 Jun 5;27(3):e14090. doi: 10.1111/hex.14090 (PMC11150858; doi:10.1111/hex.14090)
Supplement: Supplementary file 1 — Supporting information. [file HEX-27-e14090-s001.docx]

Focus group topic guide: Pain Phenotypes and their Underlying Mechanisms in Inflammatory Arthritis (PUMIA)

Introduction

- Thank you for meeting us today and offering to take part in this study.
- I would like first to outline the study so that you are able to decide whether you wish to proceed further (recap Information Sheet). In this focus group we would like to explore two key areas: firstly, the different types of pain you experience as a result of your inflammatory arthritis and secondly, the experience of living with this pain.
- Review signed consent forms (signed in advance)
- The interview should take about 60 minutes and can be paused at any time
- Feel free to ask questions at any stage during the interview
- I might write a few notes to remind myself to ask some questions later or at the end of the focus group. If you provide information you do not wish to have recorded, I can stop the audio recorder at any time and restart with your agreement.
- Include ground rules: not speaking over each other, one at the time,
- All that is said in the focus group cannot be shared outside the meeting, as this is confidential information
- Please let me know if you are uncomfortable or need a break at any point, and feel free to get up/move around and not participate at any time if needed
- *Give each person an opportunity to talk e,g. give three examples or speak for 5 minutes each. If it takes too much time, ask people to raise their hands if they agree with a statement of one or two participants and make a note of this (eg. that x number of people agreed with the statement of one or two members)*

Interview

- - Could you tell me, each in turn, about your pain that you experience daily
  - How would you describe your pain? What does it feel like?
    - Describe the pains in more detail, Characteristics of pain, triggering/relieving factors
  - How does your pain affect your daily life?
    - Physical: Self care, physical activity levels, fatigue
    - Mental: Mood, anxiety, stress, frustration,
    - Social: Interpersonal relationships, Hobbies, work, identity
  - What would your message be to others who do not experience pain on a daily basis to allow them to understand your pain sensation better?

Closing

- Is there something anyone would like to add?
- Debrief, ask each person how they are and that they are ok leaving the focus group, if they need support ask them to stay behind and you talk to them
